# Optimal Ovulation Trigger–Oocyte Pickup Interval in Progestin-Primed Ovarian Stimulation Protocol: A Retrospective Study Using Propensity Score Matching

**DOI:** 10.3389/fendo.2019.00694

**Published:** 2019-10-15

**Authors:** Xi Shen, Hui Long, Wenya Guo, Hongyuan Gao, Renfei Cai, Wei Jin, Zhiguang Yan, Shaozhen Zhang, Yun Wang, Qifeng Lyu, Li Wang, Yanping Kuang

**Affiliations:** The Department of Assisted Reproduction, Shanghai Ninth People's Hospital Affiliated to Shanghai Jiao Tong University School of Medicine, Shanghai, China

**Keywords:** ovulation trigger–OPU interval, progestin-primed ovarian stimulation (PPOS) protocol, mature oocyte rate, implantation rate, live birth rate

## Abstract

**Background:** To investigate the optimal ovulation trigger–oocyte pickup (OPU) interval of a progestin-primed ovarian stimulation (PPOS) protocol.

**Method:** Patients with normal ovarian reserve in their first PPOS OPU cycle were enrolled in this retrospective cohort study between July 2013 and April 2018. This retrospective cohort study included two parts. In part I, we studied the regression trend of mature oocyte rate, implantation rate, and live birth rate within the whole ovulation trigger–OPU interval of 7,258 patients. To homogenize some clinical characters that were key regulators of OPU time, in part II, we used propensity score matching to auto-select patients among trigger–OPU interval group 1 (35.6–36.4 h), group 2 (36.4–37.1 h), and group 3 (37.1–37.8 h) and analyzed clinical outcomes.

**Results:** Study part I showed that the whole ovulation trigger–OPU interval (33–39.5 h) of PPOS protocol had a trend of a high mature oocyte rate (>80%), increasing implantation rate, and high live birth rate. Propensity score matching of patients with homogeneous clinical characteristics further indicated that the trigger–OPU interval within groups 2 and 3 (36.4–37.8 h) had significantly higher mature oocyte rates (84.54% vs. 84.60% vs. 82.34%, *P* = 0.002) and implantation rates (34.17% vs. 34.37% vs. 29.61%, *P* < 0.05) than group 1. The same tend was observed in the live birth rate.

**Conclusions:** The ovulation trigger–OPU interval of 36.4–37.8 h is optimal for most patients using a PPOS protocol.

## Introduction

The interval from trigger to oocyte pickup (OPU) is the period of *in vivo* oocyte maturation that has a predominant effect on assisted reproductive technology success. This interval is crucial because a number of indispensable processes including the start of luteinization, the expansion of the cumulus cells, and the resumption of the reduction division of the oocyte should be well-established before aspiration (1). Some studies have found that a longer interval to OPU may produce more mature oocytes (1, 2) or achieve a higher fertilization rate (3). Some studies have shown that prolonging the interval between human chorionic gonadotropin (hCG) priming and oocyte retrieval could increase the proportion of MII oocytes but not pregnancy rates (4, 5). Therefore, the exploration of the optimal interval of controlled ovarian hyperstimulation (COH) protocols would benefit clinic outcomes.

Since 2013, medroxyprogesterone acetate (MPA) has been an effective oral alternative for preventing a premature luteinizing hormone (LH) surge in women undergoing COH, termed the progestin-primed ovarian stimulation (PPOS) protocol (6–9). A new COH protocol, PPOS has proven effective for patients with a normal response, diminished ovarian reserve, polycystic ovarian syndrome, and high body mass index (BMI) (6, 8, 10, 11). It has a different mechanism, hypothalamic action, to prohibit the premature LH surge than other COH protocols (12). The different follicular hormonal environment in the PPOS protocol may lead to a diversity of cytokines in the follicular fluid, which may influence the optimal trigger–OPU interval (13). Although it is popular among patients and has a promising clinical application, no studies have focused on whether different trigger–OPU intervals will influence oocyte performance and pregnancy rates of the PPOS protocol nor have they identified the optimal interval.

Therefore, this study aimed to explore the optimal ovulation trigger–OPU interval of the PPOS protocol that is universally used in our center, using a retrospective analysis of a huge number of patients. Propensity score matching (TriMatch) was used to reduce bias and enable focus on the trigger–OPU interval among patients with normal ovarian reserve and normal response. This study will offer valuable guidance to other clinics worldwide using the PPOS protocol.

## Materials and Methods

### Study Setting and Patients

This retrospective study was conducted between July 2013 and April 2018 in the Department of Assisted Reproduction of the Ninth People's Hospital affiliated with Shanghai Jiao Tong University School of Medicine. The study protocol was approved by the Ethics Committee (institutional review board) of the Ninth People's Hospital of Shanghai (no. 2014-031). Informed written consent was obtained from all individual participants included in the study. The following inclusion criteria were applied: age <40 years, in the first *in vitro* fertilization/intracytoplasmic sperm injection (IVF/ICSI) cycle using the PPOS protocol, basal follicle stimulating hormone (FSH) level <10 mIU/mL, and antral follicle count (AFC) > 5. Infertility was due to female, male, combined, or unknown factors. Study exclusion criteria were: (1) endometriosis or polycystic ovarian syndrome, (2) receipt of hormone treatments within the previous three-month period, and (3) any contraindications to ovarian stimulation treatment.

### Progestin-Primed Ovarian Stimulation (PPOS) Protocol

Briefly, the COH regimen is as follows (6): In the PPOS protocol, human menopausal gonadotropin (hMG) 150–225 IU/d (Fengyuan Pharmaceutical Co., Maanshan, China) and MPA 10 mg/d (XianJu Pharmaceutical Co., Taizhou, China) were administered from menstruation cycle days 2–5. The doses were adjusted according to transvaginal ultrasound examination findings. When three dominant follicles reached at least 18 mm or one dominant follicle reached at least 20 mm in diameter, final oocyte maturation was triggered using Triptorelin 0.1 mg (Ferring Pharmaceuticals, Germany) and/or hCG 1000 IU (Lizhu Pharmaceutical Trading Co., Shanghai, China); the default trigger time was 10 pm. A 10-min error was permitted because of drug preparation. The exact trigger time was recorded by the surgical nurses.

### Oocyte Retrieval Operation

The patients would receive a blood test at 8 a.m. on the day after the trigger. Two experienced doctors created an oocyte retrieval schedule based on every half hour (e.g., 9–9.30 a.m.) for patients at 3 p.m. according to each patient's estrogen level on the trigger day, estrogen levels on the day after the trigger day, number of follicle diameters > 14 mm and > 10 mm, age, AFC, etc. The detailed rules were as follows: (1) If the E_2_ level on the day after the trigger day decreased compared to the trigger day, the patients were scheduled to retrieve oocytes earlier than 35.5 h (mainly 34.5–35.5 h); otherwise, it was more than 35.5 h (mainly 35.5–38 h). (2) For the patients in the same trigger–OPU interval group, those with a fewer numbers of follicles > 10 mm and > 14 mm would be scheduled to retrieve oocytes earlier than those with more follicles. (3) If some patients had similar numbers of follicles > 10 mm and > 14 mm in the same group, the patients with older age or fewer AFC were scheduled for earlier oocyte retrieval.

Oocyte aspirations were performed by one of six skilled physicians in our center according to the oocyte retrieval schedule prepared 1 day prior. The timing of oocyte aspiration was defined as the midpoint of the start time (patient transferred to the operating table) and end time (patient removed from the operating table) and recorded by the surgical nurse. Very few surgeries would be postponed due to special circumstances. All available follicles > 10 mm in diameter were aspirated under transvaginal ultrasound guidance at 21–24 kPa.

### Insemination and Embryo Culture

The aspirated oocytes were transferred to the embryology laboratory in a modified HTF medium (Irvine Scientific, USA) and transferred to a culture medium. Fertilization was performed via IVF or ICSI depending on semen parameters (14). For IVF, fertilization was conducted after 4–6 h and the maturity was examined the day after the retrieval day. For ICSI, oocytes were usually preincubated for 2–3 h and then denuded and examined for maturity. After another 2 h, the immature oocytes were examined again and all of the mature oocytes were injected (15, 16).

All the embryos were cultured in separate microdroplets (continuous single culture medium; Irvine Scientific) in a humidified atmosphere containing 5% O_2_ and 5% CO_2_. Third-day embryos were evaluated according to the Cummins standard (17). The 3-day good-quality embryos were defined as grade 1 and 2 embryos with at least 8 cells (6, 17). Non-top-quality embryos were placed in an extended culture until they reached the blastocyst stage. Blastocysts with good morphology and good-quality embryos after cleavage were defined as viable embryos that could be used for frozen embryo transfer.

### Embryo Transfer and Luteal Support

Modified natural cycle or hormone replacement treatment was performed according to individual conditions (18). Briefly, a modified natural cycle was recommended for patients with a regular menstrual cycle using hCG 5000 IU (Lizhu Pharmaceutical Trading Co.) as the trigger. Artificial cycles were applied in those patients with irregular menstrual cycles or a history of abnormal uterine bleeding. Femonston tablets 4 mg/d (E_2_ tablets, Abbott Healthcare Products B.V., USA) were administered for 14 days, at which time progestin was added for luteal support. A maximum of two embryos were transferred. Moreover, Femoston tablets 4 mg/d (complex packing estradiol tablets/estradiol and dydrogesterone tablets; Abbott Healthcare Products B.V.) and soft vaginal progesterone capsules 400 mg/d (Utrogestan; Laboratories Besins-Iscovesco, Belgium) were used as progestin supplementation until 10 weeks of gestation.

### Statistical Analysis

In study part I, locally estimated scatterplot smoothing (Loess) was adjusted to relate the percentage of the oocyte maturity trends over the lag time interval of oocyte aspiration. A logistic regression model was performed to explore the adjusted implantation rate and live birth rate per embryo transfer with oocyte aspiration time. First, a binary logistic regression model analysis was performed to determine the confounding factors. Next, the significant variables (*P* < 0.1) identified on the univariate analysis were subjected to multivariate logistic regression analysis. Therefore, the implantation rate was adjusted for age, infertility duration, primary infertility, BMI, E_2_ level on trigger day, ratio of E_2_ on the day after the trigger day/E_2_ on the trigger day, and number of follicles > 14 mm. The live birth rate was adjusted for age, infertility duration, primary infertility, fertilization method, and transfer embryo number. These definitions were used: mature oocyte rate = number of mature oocytes/number of oocytes retrieved. Implantation rate = number of gestational sacs observed/number of embryos transferred. Live birth rate per transfer = number of deliveries with at least one live birth/one embryo transfer cycle (19). Live births were defined as those occurring at least 22 gestational weeks or at least 500 g.

In study part II, we included only patients whose OPU interval was between the 10th percentile and the 90th percentile [35.6–37.8 h] to reduce bias caused by the small number of patients with an OPU interval shorter than the 10th percentile (36.5 h) or longer than the 90th percentile (37.8 h). We divided them equally into three groups: group 1 (35.6 ≤ time < 36.4), group 2 (36.4 ≤ time < 37.1), and group 3 (37.1 ≤ time ≤ 37.8). To control the confounding factors of OPU time, a TriMatch analysis was performed within the three groups using R software (20); the matched ratio was 1:1:1. To determine the parameters crucial to the trigger–OPU interval, Pearson or Spearman corrections were applied between the trigger–OPU interval and other basic clinic parameters. The following parameters were chosen for the TriMatch: age, BMI, AFC, basal FSH, duration of gonadotropin (Gn) use, number of follicles > 10 mm and > 14 mm in diameter on the trigger day, E_2_ level on the trigger day, and E_2_ level on the day after the trigger day.

Statistical analyses were performed using SPSS version 22 (SPSS Inc., Chicago, IL, USA) and R software (R for Windows version 3.6.0). Variables are expressed as mean ± SD in tables and were tested with one-way analysis of variance. Qualitative data are presented as percentages and were tested with the chi-squared test or Fisher's exact test when appropriate, while the Bonferroni method was used in *post-hoc* analysis. Statistical significance was defined as a comparison resulting in *P* < 0.05.

## Results

### Study Part I: Regression Trend of Mature Oocyte Rate, Implantation Rate, and Live Birth Rate per Transfer Within Trigger–OPU Interval in Patients With Inconsistent Clinic Characteristics

**Figure 1:**
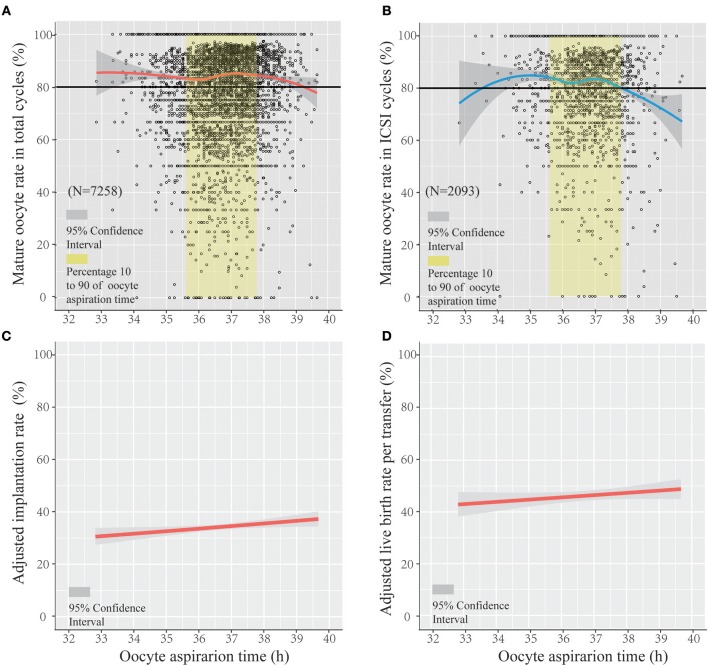
The regression trend of mature oocyte rate, implantation rate, and live birth rate per transfer within the trigger–oocyte pickup (OPU) interval of the progestin-primed ovarian stimulation (PPOS) protocol. Scatterplot of the PPOS protocol demonstrating the mature oocyte rate as a function of lag time from ovulation trigger to ovum aspiration in the total IVF/ICSI cycles **(A)** and only ICSI cycles **(B)**, featuring loess trend with the 95% confidence interval highlighted in gray. The crude and adjusted implantation rate (adjusted by age, infertility duration, primary infertility, BMI, E_2_ level on trigger day, and ratio of E_2_ on the following day after trigger/E_2_ on the trigger day and number of follicles >14 mm) and adjusted live birth rate per transfer (adjusted by age, infertility duration, primary infertility, fertilization method, and transfer embryo number) in IVF/ICSI cycles are separately shown in **(C,D)**.

Figures 1A,B show a scatterplot of mature rate on the y-axis vs. lag time on the x-axis for the total IVF/ICSI cycles (*n* = 7,258) and in only ICSI cycles (*n* = 2,093). The whole ovulation trigger–OPU interval (33–39.5 h) of the PPOS protocol shows a regression trend with a high mature oocyte rate (>80%). Figure 1A shows that the mature oocyte rate mainly stabilized at approximately 83% from 32 to 35.8 h and then slightly increased up to 85% at 37.2 h; thereafter, the mature oocyte rate gradually decreased. Figure 1B shows the trend in patients treated with the ICSI method only, in which the oocyte maturity was assessed 2–3 h after retrieval and was more accurate. The same trend was observed in the percentage of oocyte maturity in ICSI with the total IVF/ICSI.

Figure 1C shows the regression trend between the adjusted implantation rate and oocyte aspiration time (adjusted for age, infertility duration, primary infertility, BMI, E_2_ level on trigger day, ratio of E_2_ on the following day after trigger/E_2_ on the trigger day, and number of follicles > 14 mm). Figure 1D shows the adjusted live birth rate per transfer (adjusted for age, infertility duration, primary infertility, fertilization method, and transfer embryo number) using logistic regression. Figures 1C,D show that the adjusted implantation rate increased with the lag time of the trigger–OPU interval (odds radio [OR] and 95% confidence interval [CI], 1.045 [1.006–1.085], *P* < 0.05). The adjusted live birth rate per transfer increased along with trigger–OPU interval; it was not significantly related to the trigger–OPU interval (OR, 1.036 [95% CI, 0.989–1.084], *P* > 0.05).

### Study Part II: Using Propensity Score Matching to Auto-Select Patients With Homogenous Clinical Characteristics Among Three Trigger–OPU Interval Groups

In study part II, we included patients whose OPU interval was between the 10th percentile and the 90th percentile (range, 35.6–37.8) to abandon the marginal time (<10th, >90th), which had few patients and might have introduced bias to the analysis. To analyze the optimal trigger–OPU interval in the PPOS protocol, we divided this trigger–OPU interval equally into three groups: 35.6 ≤ time < 36.4 (group 1, *n* = 1,621), 36.4 ≤ time < 37.1 (group 2, *n* = 2,318) and 37.1 ≤ time ≤ 37.8 (group 3, *n* = 1,716). Although a high mature oocyte rate (>80%) and increasing implantation rate trend were seen within the whole trigger–OPU interval, including these three trigger–OPU intervals (Figure 1), the actual oocyte retrieval could not be performed in the clinic within such a long period since the patients' clinical characteristics were different. These inhomogeneous clinic characteristics were key regulators of OPU time and the confounding factors influencing the mature oocyte rate. Therefore, we used propensity score matching to auto-match patients with homogenous clinical characteristics among the three trigger–OPU interval groups.

**Figure 2:**
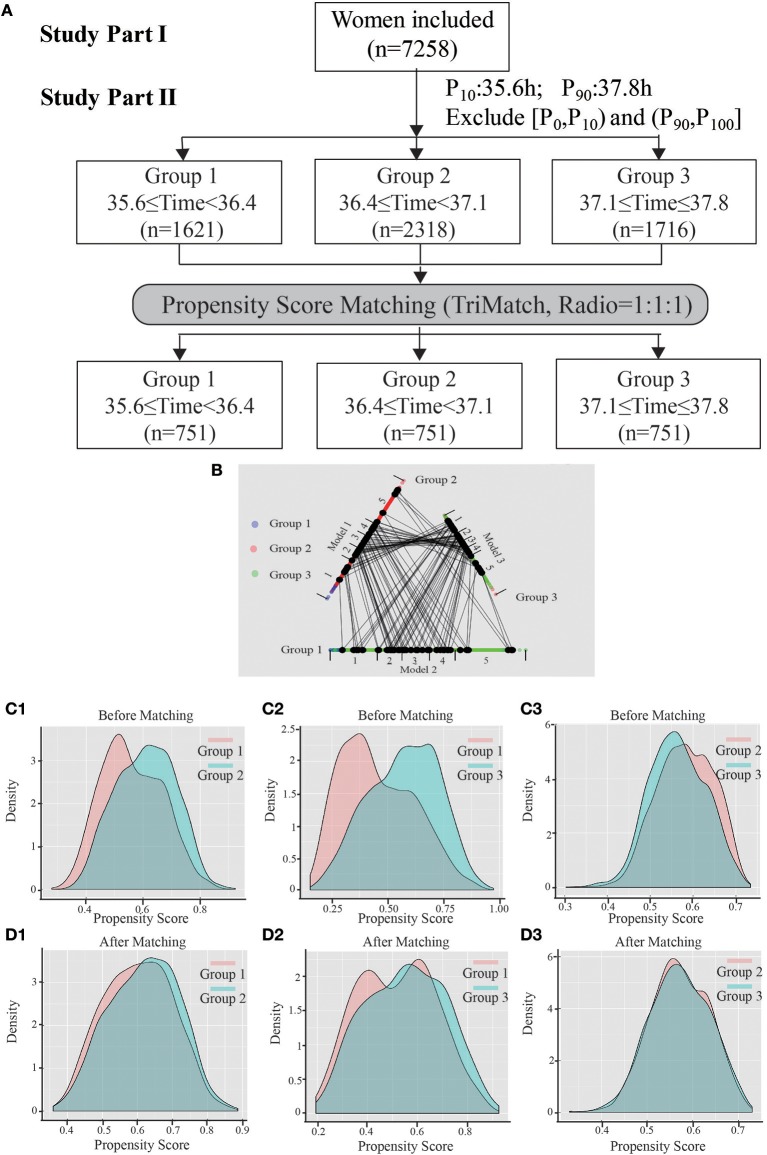
The entire study process. **(A)** Shows the flow chart, including study parts I and II. **(B)** Shows a triangle plot of matching in study part II. **(C1)** (group 1 vs. group 2), **(C2)** (group 1 vs. group 3), and **(C3)** (group 2 vs. group 3) represent the distributions of propensity scores before matching. **(D1–D3)** Show the balance after matching between cohorts.

A flow chart of the whole study is shown in Figure 2A. A triangle plot of the tri-matching procedures is shown in Figure 2B. The densities of the propensity score before and after matching are shown in Figures 2C,D. Before tri-matching, there were significant differences in the patients' clinical characters among the three groups (Table 1). After propensity score matching, no significant differences were found in the basic and clinical characteristics that were closely related with the trigger–OPU interval, including age, BMI, AFC, basal FSH, duration of Gn use, number of follicles > 10 mm and > 14 mm in diameter on the trigger day, E_2_ level on the trigger day, and E_2_ on the day after the trigger day (Table 2). Other baseline characteristics including primary infertility, infertility reasons, ovulation trigger method, and Gn dose did not differ among the three trigger–OPU interval groups (Table 2, *P* > 0.05).

**Table 1:** Features of the TriMatch cohort before matching.

**Parameters**	**Group 1** **35.6≤Time <36.4** **(hour)**	**Group 2** **36.4≤Time <37.1** **(hour)**	**Group 3** **37.1≤Time≤37.8** **(hour)**	***P*-value**
Number of patients (*n*)	1,621	2,318	1,716	
Age (year)	31.73 ± 3.81^a^	31.19 ± 3.71^b^	30.86 ± 3.66^c^	<0.001
Antral follicle count (*n*)	11.70 ± 4.89^a^	12.94 ± 4.80^b^	13.94 ± 5.06^c^	<0.001
Body mass index (kg/m^2^)	21.33 ± 3.81	21.47 ± 3.50	21.33 ± 3.56	0.349
Basal FSH (mIU/ml)	5.79 ± 1.28 ^a^	5.62 ± 1.26 ^b^	5.50 ± 1.23 ^c^	<0.001
No. of follicles with diameter > 10 mm on trigger day (*n*)	11.58 ± 6.27 ^a^	14.28 ± 6.88 ^b^	15.84 ± 6.89 ^c^	<0.001
No. of follicles with diameter> 14 mm on trigger day (*n*)	8.34 ± 5.63^a^	10.36 ± 6.35^b^	11.50 ± 6.30^c^	<0.001
E_2_ level on the trigger day (pg/ml)	2884.86 ± 1423.21^a^	3435.01 ± 1359.14^b^	3739.12 ± 1309.59^c^	<0.001
E_2_ level on the day after trigger day (pg/ml)	3254.48 ± 1411.89^a^	3881.89 ± 1251.66^b^	4172.01 ± 1134.53^c^	<0.001
Duration of Gn use(d)	8.94 ± 1.39^a^	9.12 ± 1.33^b^	9.16 ± 1.40^b^	<0.001

*The different superscript alphabets of a, b, c stand for a significant difference (Bonferroni method)*.

**Table 2:** Features of the TriMatch cohort after matching.

**Parameters**	**Group 1** **35.6≤Time <36.4 (hour)**	**Group 2** **36.4≤Time <37.1 (hour)**	**Group 3** **37.1≤Time≤37.8 (hour)**	***P*-value**
Number of patients (*n*)	751	751	751	
Age (year)^*^	31.10 ± 3.76	31.29 ± 3.62	31.33 ± 3.60	0.417
Body mass index (kg/m^2^)^*^	21.50 ± 2.96	21.30 ± 2.58	21.31 ± 3.18	0.343
Primary infertility n(%)	446 (59.39)	421 (56.06)	437 (58.19)	0.442
Antral follicle count (n)^*^	13.13 ± 5.26	12.80 ± 4.63	13.04 ± 4.80	0.404
Basal FSH (mIU/ml)^*^	5.60 ± 1.23	5.61 ± 1.25	5.68 ± 1.28	0.451
Infertility reasons *n* (%)				0.895
Female factors	436 (58.06)	426 (56.72)	431 (57.39)	
Male factors	132 (17.58)	119 (15.85)	128 (17.04)	
Combined	70 (9.32)	81 (10.79)	75 (9.99)	
Others	113 (15.05)	125 (16.64)	117 (15.58)	
Duration of Gn use (d)^*^	9.07 ± 1.23	9.02 ± 1.25	9.13 ± 1.45	0.318
Gn doses (IU)	1992.24 ± 319.03	1988.50 ± 323.52	2007.32 ± 379.47	0.528
No. of follicles with diameter>10 mm on trigger day(*n*)^*^	13.79 ± 6.41	13.81 ± 6.35	13.91 ± 6.10	0.925
No. of follicles with diameter>14 mm on trigger day (*n*)^*^	9.94 ± 6.06	9.97 ± 5.83	10.00 ± 5.37	0.984
E2 level on the trigger day (pg/ml)^*^	3377.08 ± 1340.31	3418.20 ± 1329.33	3368.85 ± 1358.06	0.747
E2 level on the day after trigger (pg/ml)^*^	3759.52 ± 1276.49	3880.74 ± 1222.74	3882.86 ± 1235.76	0.090
Ovulation trigger method				0.962
GnRHa	29 (3.86)	26 (3.46)	26 (3.46)	
hCG	40 (5.33)	38 (5.06)	35 (4.66)	
Dual trigger	682 (90.81)	687 (91.48)	690 (91.88)	

* *Represents the parameters were used for propensity score matching; the different superscript alphabets of a, b stand for a significant difference (Bonferroni method)*.

### Evaluating Optimal Trigger–OPU Interval of PPOS Protocol From Mature Oocyte Rate and Implantation Rate

**Table 3** demonstrated no significant differences in the number of retrieved oocytes (12.02 vs. 12.06 vs. 11.76, *P* > 0.05) and mature oocytes (9.80 vs. 10.06 vs. 9.86, *P* > 0.05). However, the trigger–OPU interval of group 2 (84.54%) and group 3 (84.60%) had significantly higher mature oocyte rates than group 1 (82.34%, *P* = 0.002). Additionally, the same trend was found in the proportion of cycles with more than 70% mature oocytes among the three trigger–OPU interval groups (82.69% vs. 83.75% vs. 77.63%, *P* = 0.009).

**Table 3:** Oocyte performance and pregnancy outcomes in TriMatch cohort.

**Parameters**	**Group 1** **35.6≤Time <36.4 (hour)**	**Group 2** **36.4≤Time <37.1 (hour)**	**Group 3** **37.1≤Time≤37.8 (hour)**	***P*-value**
Oocyte retrieved (*n*)	12.02 ± 6.61	12.06 ± 6.83	11.76 ± 6.58	0.640
MII oocyte (*n*)	9.80 ± 5.80	10.06 ± 5.87	9.86 ± 5.68	0.655
Mature oocyte rate (%)	82.34 ± 18.85 ^a^	84.54 ± 17.26 ^b^	84.60 ± 18.20 ^a, b^	0.002
Cycles with >70% mature oocytes (*n*) (%)	583 (77.63)^a^	621 (82.69)^b^	629 (83.75)^b^	0.009
Insemination methods *n* (%)				0.380
IVF	420 (55.93)	442 (58.85)	430 (57.26)	
ICSI	220 (29.29)	224 (29.83)	226 (30.09)	
IVF+ICSI	111 (14.78)	85 (11.32)	95 (12.65)	
Normal fertilized oocyte (*n*)	8.21 ± 5.18	8.26 ± 5.26	7.97 ± 5.08	0.524
Viable embryos (*n*)	4.19 ± 2.90	4.34 ± 3.21	4.01 ± 3.13	0.105
No. of embryo transfer cycles (*n*)	1,016	939	951	
Thickness of endometrium (mm)	10.15 ± 2.19	10.14 ± 2.17	10.28 ± 2.29	0.323
Endometrium preparation protocol n (%)				0.277
Modified natural cycle	688 (67.72)	652 (69.44)	678 (71.29)	
Artificial cycle	328 (32.28)	287 (30.56)	273 (28.71)	
Implantation rate (%)	29.61 (551/1861)^a^	34.17 (585/1712)^b^	34.37 (588/1710)^b^	0.003
Live birth rate per transfer (%)	39.57 (402/1016)	43.56 (409/939)	42.06 (400/951)	0.194

*The different superscript alphabets of a, b stand for a significant difference (Bonferroni method)*.

There were no significant differences in normal fertilized oocytes (8.21 vs. 8.26 vs. 7.97, *P* > 0.05) and viable embryos (8.21 vs. 8.26 vs. 7.97, *P* > 0.05). However, we found that the best embryonic performance was observed with the trigger–OPU interval of group 2. Regarding pregnancy outcomes, the implantation rates differed significantly among the three groups. The trigger–OPU interval of group 2 (34.17%) and group 3 (34.37%) had significantly higher implantation rates than group 1 (29.61%, *P* < 0.05). Moreover, the superiority of the implantation rates of groups 2 and 3 was passed into the final live birth rate. The trigger–OPU interval of group 2 (43.56%) and group 3 (42.06%) had higher live birth rates per transfer than group 1 (39.57%, *P* > 0.05), but there were no significant differences. Furthermore, we analyzed the implantation rate and live birth rate of the first frozen embryo transfer (FET) cycle. The implantation rate (29.48% vs. 35.62% vs. 34.04%, *P* < 0.05) and live birth rate (39.94% vs. 47.54% vs. 44.22%, *P* < 0.05) were significantly higher in groups 2 and 3 than in group 1. These results further verified that the trigger–OPU intervals of groups 2 and 3 had better pregnancy outcomes than group 1 in the first transfer cycle.

## Discussion

This study first explored the optimal trigger–OPU interval of PPOS protocol mainly according to the mature oocyte rate. In study part I, a high and stable trend of mature oocyte rate (>80%) was observed within whole trigger–OPU time periods (33.0–39.5 h in IVF/ICSI) of the PPOS protocol, and an increasing adjusted implantation rate and live birth rate were also observed. In study part II, we further explored the optimal trigger–OPU interval of the PPOS protocol within a 35.6–37.8 h time period when 80% oocyte retrieving was concentrated. The patients in trigger–OPU interval groups 2 and 3 (36.4–37.8 h) with homogenous clinic characteristics, which were auto-selected by propensity score matching, had significant higher mature oocyte rates and implantation rates than those in group 1. The same trend was observed in the live birth rate.

### Strengths and Limitations

This study first explored the optimal trigger–OPU interval of the PPOS protocol considering the mature oocyte and implantation rates. PPOS is a new, widely concerned COH protocol (9) that was well-received by patients in our center and showed a promising application and has a different mechanism for preventing LH surge, mainly from hypothalamic suppression (12). The optimal trigger–OPU interval of the PPOS protocol has its own feature that requires exploration. Since 2013, more than 7,000 normal ovarian reserve patients have used this protocol in our center. As its initiator, we had a large number of patients and accumulated much experience with trigger–OPU intervals. Therefore, this study first analyzed the optimal ovulation trigger–OPU interval in normal ovulary women treated with the PPOS protocol, providing enlightenment for clinical practice worldwide.

We used propensity score matching analysis to auto-match the patients with similar age, ovarian reserve, dosage and duration of Gn use, ovarian response, number of follicles with a diameter > 14 or > 10 mm on the trigger day, E_2_ level on the trigger day, and E_2_ level on the day after the trigger day among the three trigger–OPU interval groups (35.6–36.4, 36.4–37.1, and 37.1–37.8, respectively) to analyze the optimal trigger–OPU interval in the PPOS protocol, primarily using the mature oocyte and implantation rates. The propensity score matching method is useful in observed retrospective studies in which treatment allocation is non-random and can be viewed as an approach seeking to replicate the random assignment in conventional randomized controlled trials (21). When homogenizing these clinical characteristics in superovulation by propensity score matching, the differences in mature oocyte and implantation rates were most likely caused by oocytes retrieved within different trigger–OPU intervals. The following analysis of the optimal trigger–OPU interval of the PPOS protocol regarding mature oocyte rate and implantation rate was meaningful and instructive.

The main limitation of this study is its retrospective nature without randomization. Second, we mainly focused on patients with similar ovarian reserves and responses (Table 2), which represents only a portion of infertility patients. This optimal trigger–OPU interval in the PPOS protocol may be not suitable for poor ovarian response, PCOS, or other peculiar patients. For poor ovarian response patients, doctors tend to schedule those patients with fewer follicles to an earlier part (e.g., 35–36 h) due to their decreasing E_2_ concentrations and higher cancellation rates (22). At the same time, we tend to schedule PCOS patients for relatively later parts (e.g., 37 h), which is necessary for oocyte maturation in PCOS patients (23). Furthermore, because very few patients (about 0.1% of normal responders) could not undergo oocyte retrieval on the retrieval day, or significantly fewer oocytes were retrieved than expected in clinical practice, we should focus on these types of patients and think about rescheduling their trigger–OPU interval in the next cycle according to their cycle characteristics. Third, there were three types of ovulation trigger methods in our center, but the proportion of gonadotropin releasing hormone (GnRH) agonist or hCG trigger was very small. We occasionally use GnRH agonists in patients with a higher possibility of ovarian hyperstimulation syndrome as well as hCG in patients with a very low LH level during COH as they might not reactive to the GnRH agonist trigger. Therefore, well-designed and adequately powered randomized controlled trials (RCTs) are needed to verify our finding in future research. Moreover, the marginal interval should be accessed in future RCTs to reduce bias.

### Comparisons of Results of Previous Studies With Those of Ours

As a new stimulation regimen specially reported by Nathalie Mass in Human Reproduction Update (9), the PPOS protocol had its differentiated action site and mechanism in prohibiting premature ovulation. The Progestin inhibits E_2_ positive feedback and abolishes the preovulatory GnRH and gonadotrophin surge when administered before or concurrent with E_2_ (12, 24). Moreover, progesterone's inhibitory effect on the GnRH/LH surge is mediated by its receptor in the hypothalamus (12). Meanwhile, lipidomic components alterations of human follicular fluid were found in PPOS patients (13). Therefore, it is necessary to explore the optimal oocyte retrieval interval of a new COH protocol with a distinct mechanism and endocrine profile.

Propensity score matching (PSM) has its advantage in the retrospective analysis of optimal trigger–OPU interval in COH protocols; the patients who were auto-matched by this method had the similar basic and clinic characteristics in superovulation, which let the analysis focus on the trigger–OPU interval and reduce other confounders. This is the first research adopting this method to analyze the optimal trigger–OPU interval of one COH protocol, which may make the analysis more meaningful. Furthermore, there were 751 patients with nearly 1,000 transferred FET cycles in each time group. The presence of a large number of patients after propensity score matching make this retrospective analysis more reliable. Therefore, the propensity score matching and large sample size make the optimal trigger–OPU interval of the PPOS protocol more instructive in the clinic when facing patients with similar characters as in Table 2.

We evaluated the optimal trigger–OPU interval of the PPOS protocol mainly from the mature oocyte rate, which was the one generally used in most previous articles regarding trigger–OPU interval (1, 4, 5, 23). We found that group 2 (36.4–37.1 h, 84.54%) and group 3 (37.1–37.8 h, 84.60%) had significantly higher mature oocyte rate than group 1 (35.6–36.4 h, 82.34%, *P* = 0.002), indicating the prolonged oocyte retrieval interval is beneficial for oocyte maturity in the PPOS protocol. Furthermore, we confirmed the optimal trigger–OPU interval of the PPOS protocol from the implantation rate, which could directly reflect the quality of each transferred embryo (25, 26). The implantation rate per embryo transferred was also significantly higher in group 2 (34.17%) and group 3 (34.37%) than in group 1 (29.61%, *P* < 0.05), which supported that a longer trigger–OPU interval was beneficial for oocyte maturity and developmental potential. Moreover, the final live birth rate per transfer further verified that the trigger–OPU interval within group 2 and 3 (36.4–37.8 h) was optimal for the PPOS protocol. Further analysis in the 1st FET cycle also demonstrated that the trigger–OPU interval groups 2 and 3 had better pregnancy outcomes.

In addition, we explored the optimal ovulation trigger–OPU intervals not only in IVF/ICSI, but also in ICSI. In our center, the interval between oocyte retrieval and oocyte maturation assessment is different in IVF and ICSI. In ICSI, oocyte maturity was examined on the retrieval day. In IVF, the oocytes were preincubated 4–6 h after retrieval and then inseminated, and the maturity was examined on the day after the retrieval day. Therefore, oocyte maturity in ICSI is much closer to the time of OPU, and it is important and necessary to evaluate the optimal ovulation trigger–OPU interval. In our research, a similar trend was found in only ICSI cycles (Figure 1). The relationships were different in the IVF/ICSI and ICSI cycles when the lag time was very early or late (two terminals in the x-axis), which may be due to the small number of patients in the ICSI cycles. In study part II, we included patients whose OPU interval was between the 10th percentile and the 90th percentile (35.6–37.8 h) due to the small sample of patients shorter than 10th percentile (36.5 h) and longer than 90th percentile (37.8 h). Another reason is that the two marginal intervals mentioned above may lead to some biases. For example, the patients who had sharp fall in E_2_ level were usually in the <35.6 h group, which would lead to a higher cancelation rate (21), and, if there were too many patients receiving an OPU operation at the same day (e.g., more than 60 patients), few patients' OPU operation would be postponed (>37.8 h).

### Possible Mechanisms of the Individual Optimal Ovulation Trigger–OPU Intervals in PPOS Protocol

The PPOS protocol has its own optimal trigger–OPU interval, which is between 36.4 and 37.8 h with better mature oocyte rate, implantation rate, and live birth rate per transfer. Some studies reported that better oocyte retrieval time varied in long protocol, such as 35–38 h (5), >36 h (4), 35–36 h (27), and 38–41 h (28). As far as natural cycle or mild stimulation cycle are concerned, the shorter interval was reported, such as 32–36 h (29) and 35–36 h (30). For the GnRH antagonist protocol, oocyte aspiration should be scheduled between 36 and 37 h without compromising results (27). Thus, it can be seen that the different COH protocols, which have diverse effects of superovulation and mechanisms of prohibiting premature LH surge, give rise to the discrepancies in the optimal trigger–OPU intervals.

The PPOS protocol has a relatively longer time period in which to achieve high mature oocyte rates, implantation rates, and live birth rates among three ovulation trigger–OPU interval groups (Table 3). The hMG and progestin are administrated together from menstrual cycle days 2–5 onward in our PPOS protocol (6, 31), which brought the hypothalamic suppression at the beginning of ovarian hyperstimulation. The LH values gradually decrease during ovarian stimulation, and the average LH level on the trigger day is significantly lower than the basal LH value, which indicates that the PPOS protocol could powerfully suppress the preovulatory LH surge. This potent hypothalamic suppression and consequent inhibition of LH level may be an important factor in prolonging the ovulation trigger–OPU interval in the PPOS protocol compared with other protocols, such as natural cycles or mild stimulation protocols. Furthermore, a high progesterone level from menstruation cycle day 3 onward may also influence the follicles. Progesterone inhibits membrane-bound adenylate cyclase, the activity of which may require more time to recover after the trigger (32), which may explain why the lower mature oocyte rate was observed in group 1.

## Conclusion

In our initiated PPOS protocol, the whole trigger–OPU interval (33–39.5 h) had a regression trend of a high and stable mature oocyte rate, increasing the implantation rate and live birth rate. After homogenizing the clinical characteristics of patients that were key regulators of OPU interval by propensity score matching among the three trigger–OPU interval groups, we found that the longer trigger–OPU interval within group 2 (36.4–37.1 h) and group 3 (37.1–37.8 h) brought a higher mature oocyte rate, significantly increasing the implantation rate and live birth rate per transfer, indicating that 36.4–37.8 h was the optimal trigger–OPU interval for most patients using the PPOS protocol.

## Data Availability Statement

The datasets generated for this study are available on request to the corresponding author.

## Ethics Statement

The present study was a retrospective cohort study conducted at the Department of Assisted Reproduction of Shanghai Ninth People's Hospital affiliated with Shanghai Jiao Tong University School of Medicine. The study protocol was approved by the hospital's Ethics Committee of Ninth People's Hospital (Institutional Review Board) (No: 2014-031).

## Author Contributions

YK and LW supervised the entire study, including the procedures, conception, design, and completion. XS conducted the acquisition of data, analysis, and interpretation of data, and drafted the article. HL, WG, HG, RC, SZ, and YW conducted the acquisition of data and revised the article. WJ, ZY, and QL conducted data analysis. All authors approved the final version of the manuscript.

### Conflict of Interest

The authors declare that the research was conducted in the absence of any commercial or financial relationships that could be construed as a potential conflict of interest.
